# A Fat to Forget: Trans Fat Consumption and Memory

**DOI:** 10.1371/journal.pone.0128129

**Published:** 2015-06-17

**Authors:** Beatrice Alexandra Golomb, Alexis K. Bui

**Affiliations:** Department of Medicine, University of California San Diego, La Jolla, California, United States of America; University of Cologne, GERMANY

## Abstract

**Purpose:**

We sought to assess the relation of dietary trans fatty acid (dTFA) consumption to word-memory.

**Methods:**

We analyzed cross-sectional data from the 1999-2005 UCSD Statin Study. Participants were 1018 adult men and non-procreative women age ≥20 without diagnosed diabetes, CVD, or extreme LDL-cholesterol. Primary analyses focused on men, as only men (N = 694) were effectively represented in younger adult ages. “Recurrent words” assessed word memory. dTFA (grams/day) estimates were calculated from the Fred Hutchinson Food Frequency Questionnaire. Regression, stratified at age 45, assessed the relation between memory and dTFA in various adjustment models. Major findings were replicated in the full sample (including women). Potential mediators were examined.

**Results:**

An age-by-dTFA interaction was significant. dTFA adversely predicted memory in younger adults (only), robust to adjustment model. Each gram/day dTFA was associated with an estimated 0.76 fewer words recalled (full model) (SE = 0.27, 95%CI = 0.22,1.3, P = 0.006). Adjustment for systolic blood pressure, waist circumference and BMI (but not lipid or glycemic variables) attenuated the relationship, consistent with mediation by factors involving, relating to, or concurrently influencing, these factors.

**Conclusion:**

Greater dTFA was significantly associated with worse word recall in younger adults. Prooxidant and energetic detriments of dTFA and triangulation with other evidence offer prospects for causality.

## Introduction

Dietary trans fatty acids (**dTFAs**), which are primarily from industrial production[1], have been linked to adverse effects on lipid profiles, metabolic function, insulin resistance, inflammation, and cardiac and general health[2–17]. Each of these in turn has adverse associations with, and potential for adverse consequences to, cognitive function[18–22].

Additionally, long chain omega-3 fatty acids (**n3FAs**)[23–25], which are anti-inflammatory and for which production is inhibited by dTFAs[26, 27], are important to brain health with favorable central nervous system effects reported for behavior[24] and mood[24]. For those outcomes, dTFAs have shown adverse associations[28, 29]. n3FAs have been presumptively linked to favorable cognitive function[30, 31], adding further rationale for assessing for an adverse dTFA relation to memory.

Moreover, dTFAs adversely affect oxidative stress[12, 32], which has adverse consequences to cell energy. Oxidative stress promotes endothelial dysfunction (limiting adequacy of blood flow and hence delivery of energy substrates), as well as mitochondrial dysfunction (reducing production of ATP from energy substrates that are delivered)[33]. Among cognitive functions, memory may be particularly sensitive to cell energy effects. Hippocampal cells (area CA1) are selectively vulnerable to death in settings of impaired energy, such as episodes of hypoxemia, hypoglycemia, or ischemia[34–36]. Thus, oxidative-energetic effects may be expected to potentiate the impact of other insults on hippocampal cell viability, and hence memory function.

It was previously shown that more frequent consumption of chocolate—a dietary substance that is rich in antioxidants[37, 38], which enhances cerebral blood flow[39] and improves mitochondrial biogenesis[40]—was linked to better memory performance[41]. This effect was strong among younger adults, but evident only in younger adult ages, particularly age <45[41]. (Children were not assessed.)

Indeed, in the prior chocolate analysis, none of the memory predictors, including chocolate, that were significant in younger age retained significance into older adult age in cross-sectional analysis. This is despite the fact that cocoa flavanols experimentally improve cognition in older age[42]. This is likely because of twin factors that affect detection of cognitive compromise in older age. In older age, any effect of a memory-adverse exposure is superimposed on the effects of other sources of (generally downward) variability that add variance and extend the range downward, and then those whose function is below some threshold disproportionately fail to participate in studies[43]. Together these effects compromise ability to detect the association or effect of an exposure on memory, cross-sectionally, in older age. Indeed, the mean word-recall score in older participants (up to age 85 years) was closely similar to that in younger age[41], rather than markedly worse, and variance—expected to be higher where there are accrued age-related sources of loss—was actually lower.

We capitalized on baseline data from a clinical trial sample with broad participation parameters that included dietary and word memory assessments to test the conjectures that dTFAs, in contradistinction to chocolate, might be associated with worse memory performance, and that an effect, if present, may be selectively evident in younger adult ages.

## Methods

Participants were 1,018 adult men and nonprocreative women (surgically or chronologically postmenopausal), who had been screened for participation in the UCSD Statin Study. Participants were relatively broadly sampled including male and female adults age ≥20, with no restriction on ethnicity, education or occupation. However, persons on lipid medications, or with extremes of LDL-cholesterol (<115mg/dL or >190mg/dL), known diabetes, cardiovascular disease, HIV, or cancer were excluded[28][44]. Since participants were screenees for a drug trial[44, 45], women of procreative potential were excluded. For this reason, only males are well-represented in younger adult ages, thus spanning the adult age spectrum. Males are therefore the focus of the primary analysis.

The study protocol was approved by the University of California, San Diego Human Research Protections Program. All participants gave written informed consent.

### Dietary Trans Fatty Acid Estimation

645 of 694 men (93%) completed a dietary survey prior to their baseline visit, using a questionnaire developed by the Nutrition Assessment Shared Resource of the Fred Hutchinson Cancer Research Center[46]. Consumption frequency and portion size were queried for a series of food categories, each in turn defined by a series of foods or beverages. Additional questions relating to food preparation and purchasing further refine nutrient calculations (http://www.fhcrc.org/science/shared_resources/nutrition/ffq/)[28].

Calculations for trans fats and other nutrients “were performed using the Nutrient Data System for Research software version 4.03, developed by the Nutrition Coordinating Center, University of Minnesota Food and Nutrient Database (version 31, released November 2000), which added trans fatty acid values in 1998”[28]. Values for trans fats “were determined for all foods in the database (0% missing) and include individual contributions of 16:1 trans (trans-hexadecenoic acid); 18:1 trans (trans-octadecenoic acid); and 18:2 trans (trans-octadecadienoic acid), which encompasses cis-trans, trans-cis, and trans-trans forms; as well as total trans-fatty acids. The USDA table ‘Fat and Fatty Acid Content of Selected Foods Containing Trans-Fatty Acids’…was the primary source of trans-fatty acid information for assignment of values to foods in the database. Additional data sources included other nutrient databases and articles in the scientific literature containing trans-fatty acid values for US foods, using appropriate methodologies”[47]. dTFA is given in grams/day.

### Word memory assessment

In a “recurrent words” task[41, 48, 49], participants were sequentially presented a set of 104 cards each bearing a word. 82 of the cards displayed words shown for the first time in the set, while 22 cards displayed words that had been presented previously. Participants state whether each word was new (presented for the first time) or recurrent (presented previously). The score was the summed “hits” (correct responses, whether for a new or recurrent word), conforming to reported use of this test elsewhere[41, 48, 49].

### Covariates

Assessed covariates that could serve as confounders included age, exercise (times/week vigorous exercise for more than 20 minutes), education (scored 1–9), ethnicity (Caucasian vs. other), chocolate consumption (time/week, previously shown to relate to this memory measure[50]), and mood (Center for Epidemiological Studies Depression Scale—**CES-D**). Mood was significantly adversely linked to dTFAs in this sample (though the published results relatively emphasized the correlated but stronger aggression relationship)[28], and in the literature[29].

### Analyses

Descriptive statistics characterized the primary outcome, primary predictors, covariates, and assessed mediator variables, in all men and stratified at age 45 (with 164 men age <45, and 530 men age ≥45). Linear regression analyses stratified at age 45 was performed for men, the group in whom younger age was represented, with word memory as the outcome variable and dTFA as the primary predictor. Models assessed the dTFA relationship adjusted for age, then added sequentially exercise; ethnicity and education which were interrelated variables; and chocolate frequency and depression—also interrelated[51]. The full-adjustment models were run with addition of women from the sample (though fewer than 10 were under age 45 years) for qualitative similarity.

To affirm an age-interaction of relevance to the 45-year stratification in men, the fully adjusted model was repeated with addition of an age-by-dTFA interaction term, with equal years represented on each side of 45 (20–44, 45–69). (Both components of the interaction term were included in the regression.)

Analyses designed to assess potential mediators added adjustment for metabolic markers individually and in combination: HDL (mg/dL), triglycerides (mg/dL), LDL (mg/dL), glucose (mg/dL), insulin (mU/mL), waist circumference (cm), BMI (kg/m^2^), and systolic blood pressure (**SBP**) (mmHg). dTFAs have elsewhere related to these variables, and in this sample significantly predicted waist circumference and BMI[52], as well as SBP.

Analyses used Stata 8.0 and 11.0, College Station, TX. A 2-sided P-value <0.05 defined statistical significance.

## Results

**Table 1**shows participant characteristics for men, the focus of the main analysis. (Age range for men and for all participants was 20–85 years.) The average word memory score (out of a possible 104) was 85 words correctly identified as new or recurrent. The mean scores were 86 for those age <45; and 85 for those age ≥45. dTFA was higher in young men than in the remainder of the sample (mostly older, as there were few young women), P = 0.001. Mean dTFA was 3.8 grams/day overall; 4.1 in age <45, and 3.7 in age ≥45. The maximum estimated dTFA was 15.5 grams/day in men under age 45, and 27.7 for the overall sample.

**Table 1:** Sample Characteristics (Men).

Variable	All (N = 694)	Age <45 (N = 164)	Age ≥45 (N = 530)
	Mean (SD)	Mean (SD)	Mean (SD)
dTFA intake (grams/day)	3.8 (2.7)	4.1 (2.9)	3.7 (2.7)
Recurrent words (#correct)	85.4 (7.9)	86.4 (8.6)	85.1 (7.6)
Age (years)	54.8 (12.8)	37.9 (5.3)	60.0 (9.5)
Exercise (x/week)*	3.8 (3.2)	3.7 (3.2)	3.8 (3.2)
Education (scaled 1–9)†	5.9 (1.6)	5.7 (1.5)	6.0 (1.6)
Ethnicity (% Caucasian)	79.4	69.5	82.5
Mood (CES-D)	8.1 (7.2)	9.2 (7.5)	7.7 (7.1)
Chocolate frequency (x/week)	1.9 (2.4)	1.9 (2.4)	1.9 (2.4)
**Potential Mediating Variables**			
HDL (mg/dL)	47.7 (13.5)	44.4 (11.5)	48.8 (13.8)
Triglycerides (mg/dL)	137.9 (78.2)	151.9 (81.0)	133.6 (76.8)
LDL (mg/dL)	149.0 (25.3)	150.3 (28.0)	148.6 (24.4)
Glucose (mg/dL)	90.8 (8.9)	88.5 (8.8)	91.6 (8.7)
Insulin (mU/mL)	16.3 (11.4)	16.7 (8.4)	16.1 (12.2)
SBP (mmHg)	127.2 (13.5)	122.8 (9.7)	128.6 (14.2)
Waist circumference (cm)	100.3 (10.7)	98.5 (10.5)	100.8 (10.7)
BMI (kg/m^2^)	28.0 (4.0)	28.1 (4.0)	28.0 (4.1)

SD = standard deviation; dTFA = dietary trans fatty acid; CES-D = Center for Epidemiological Studies Depression Scale; HDL = high density lipoprotein cholesterol; LDL = low density lipoprotein cholesterol; SBP = systolic blood pressure; BMI = body mass index.

*Vigorous exercise >20min.

†Education scoring: 1 = no high school; 2 = some high school; 3 = high school graduate; 4 = technical school; 5 = some college; 6 = college graduate; 7 = masters degree; 8 = other postgraduate degree; 9 = doctoral degree.

Table 2 shows the relationship between dTFAs and memory performance in men age <45 years, in a range of models adjusting for potential confounders—adjusted for age only, then successively adding exercise, ethnicity and education (related to one another including in this sample), and chocolate frequency and mood (also related in this sample[51]). Trans fat consumption adversely predicted word-recall performance in younger adults age across the adjustment models.

**Table 2:** Dietary Trans Fat Relation to Memory, Stratified by Age: Men Age <45.

	dTFA Relation to Memory		
Model Adjusts For	β (SE)	95%CI	P
Age	-0.49 (0.24)	-0.97, -0.02	0.043
Age, exercise	-0.50 (0.24)	-0.99, -0.02	0.041
Age, exercise, education, ethnicity	-0.65 (0.28)	-1.20, -0.11	0.020
Age, exercise, education, ethnicity, CES-D, chocolate	-0.76 (0.27)	-1.29, -0.23	0.006

dTFA = dietary trans fatty acid; β = regression coefficient; SE = standard error; CI = confidence interval; CES-D = Center for Epidemiological Studies Depression Scale.

Results of linear regression of dTFA on recurrent word score, with adjustments as per Table. Retaining women in the analysis (N = 9 women only were age <45) does not materially alter the findings: for the fully adjusted model, β = -0.73 (SE = 0.26, 95%CI = -1.25, -0.21), P = 0.006. However there were few such women and they were constrained to be surgically or otherwise postmenopausal.

In contrast, there was no relation of dTFAs to word memory in those over age 45 (Table 3). Other predictors (chocolate, mood, ethnicity) also lost significance in older age.

**Table 3:** Dietary Trans Fat Relation to Memory, Stratified by Age: Men Age ≥45.

	dTFA Relation to Memory		
Model Adjusts For	β (SE)	95%CI	P
Age	-0.026 (0.13)	-0.28, 0.23	0.84
Age, exercise	-0.027 (0.13)	-0.28, 0.23	0.84
Age, exercise, education, ethnicity	0.022 (0.14)	-0.24, 0.29	0.87
Age, exercise, education, ethnicity, CES-D, chocolate	0.067 (0.14)	-0.20, 0.34	0.63

dTFA = dietary trans fatty acid; β = regression coefficient; SE = standard error; CI = confidence interval; CES-D = Center for Epidemiological Studies Depression Scale.

Results of linear regression of dTFA on recurrent word score, with adjustments as per Table. Retaining women in the analysis (N = 315 women were age ≥45) does not materially alter the findings: for the fully adjusted model, β = -0.072 (SE = 0.12, 95%CI = -0.31, 0.17), P = 0.55.

The primary analysis focused on men, since women under age 45 were few and were surgically or chronologically postmenopausal, and thus not reflective of their age group. Of note, however, findings did not differ materially with inclusion of women under age 45, and retained the same significance value of P<0.006 in the fully-adjusted model.

Assessing the age-interaction using the fully adjusted model affirmed a strong age-by-dTFA interaction term: per year of age and gram/day of dTFAs, interaction β = 0.028 (SE = 0.013, 95%CI = 0.004, 0.053), P = 0.025. The dTFA term was separately significant β = -1.52 (SE = 0.64; 95%CI = -2.79, -0.26), P = 0.018. This supports the age-stratified analysis.

Table 4 shows the effect of adjusting for potential mediating variables, variables that dTFAs have been reported to influence, that might serve in the causal pathway to adverse memory (or alternatively, that factors in the causal pathway might concurrently influence). Addition of variables in the causal pathway (or concurrently affected by mechanisms in the causal pathway) is expected to attenuate or obviate the association. Adjustment for HDL, triglycerides, LDL, glucose and insulin, separately or together, did not materially attenuate the relationship of dTFAs to memory. However, adjustment for SBP, waist circumference and BMI did substantially attenuate significance of the dTFA prediction. Of relevance, SBP, waist circumference and BMI did not themselves significantly predict memory performance, suggesting that mechanisms by which dTFAs affect BMI (rather than BMI itself) may also affect memory impairment, further supporting use of these terms as (*proxies for*) mediators, rather than as confounders. The relationship remained (just) significant with adjustment for these three predictors separately and together. Adjustment for all metabolic variables together did abrogate significance, but in a model that is “overadjusted” (violating the heuristic of 10 participants per predictor, with 15 predictor variables).

**Table 4:** Dietary Trans Fat Relation to Memory: Effect of Adding Potential Metabolic Mediators to Full Model (Men Age <45)*.

	dTFA Relation to Memory		
Adjustments in addition to above	β (SE)	95%CI	P
HDL	-0.75 (0.27)	-1.3, -0.22	0.006
Triglycerides	-0.72 (0.26)	-1.2, -0.19	0.008
LDL	-0.71 (0.27)	-1.2, -0.18	0.009
Glucose	-0.78 (0.27)	-1.3, -0.24	0.005
Insulin	-0.74 (0.28)	-1.3, -0.19	0.009
All lipid and glycemic variables	-0.69 (0.28)	-1.2, -0.14	0.014
SBP	-0.51 (0.26)	-1.0, -0.00	0.049
Waist circumference	-0.56 (0.26)	-1.1, -0.04	0.037
BMI	-0.56 (0.26)	-1.1, -0.04	0.034
SBP, waist, and BMI	-0.53 (0.27)	-1.1, -0.00	0.049

dTFA = dietary trans fatty acid; β = regression coefficient; SE = standard error; CI = confidence interval; HDL = high density lipoprotein cholesterol; LDL = low density lipoprotein cholesterol; SBP = systolic blood pressure; BMI = body mass index.

*Results of linear regression of dietary trans fatty acid consumption (dTFA, grams/day) on recurrent word score, adjusted for age, exercise, education, ethnicity, mood, and chocolate frequency, in addition to the adjustment variables listed.

None of the metabolic predictors were themselves significant or borderline significant in predicting word memory, except triglycerides were a positive predictor (P = 0.032 in the model adding only triglycerides; P = 0.11 with other lipid and glycemic predictors).

## Discussion

Greater dTFA consumption showed an adverse relation to word recall performance in adults age <45 years, working years in which much productive and creative work is undertaken. To our knowledge, an adverse relationship of dTFAs to memory or cognition in younger adulthood has not been previously shown. The relevance of the effect, an estimated 0.76 fewer words recalled per gram/day dTFA should be viewed in the context that participant dTFAs at the time of the study ranged up to 28 grams/day—which in a man age <45 would yield an estimated 21 fewer correct word-recall responses, in the setting of an average score of 86. (In the smaller sample of men age <45, the maximum reported consumption of 15.7 grams/day would yield an estimated 11–12 fewer correct responses. Since younger men on average had *higher* dTFA than others, P = 0.001, and higher variance, it might be expected that a higher maximum dTFA would be observed had a larger sample of young men been available.) The relationship was robust within the younger adult group to adjustment for potential confounders such as age, exercise, education, ethnicity, and mood. Younger adults were predominantly men, and the analysis focused on that group; however, including the small number of women under 45 did not modify the strong significance of the adverse dTFA relationship to word memory.

An association of dTFAs to word memory was not observed in older adults (age ≥45), consistent with expectation from findings with other predictors, and consistent with nonparticipation of more impaired participants, increasingly removing a relevant part of the distribution with increasing age[43]. (Greater variance in word memory introduced by accrued cognitive losses with age, leading the range to be extended downward into cognitive territory that may contribute to nonparticipation in studies[41], could mean the restricted visible portion of the distribution no longer retains comparable power to detect associations.) This limits implications of the study in the older age group, but does not diminish authority of observed associations in younger age.

In those age <45, in whom dTFAs adversely predicted word memory: only SBP, waist circumference, and BMI (which relates to dTFAs in humans[52] and animals[53]), among assessed potential mediators, materially attenuated significance of the relationship. Despite reducing significance of the dTFA association to memory, none independently predicted memory performance. This suggests that dTFA-related mechanisms influenced these metabolic parameters *in parallel with* cognition, rather than these metabolic variables themselves bearing a mediating role. dTFA consequences to oxidative stress and cell energy are leading candidates for this effect: triangulating evidence links oxidative stress and *adverse* cell energy deleteriously to BMI, SBP and other metabolic syndrome variables[33]. Oxidative stress is tied to cognitive decline observationally in humans[54, 55] and experimentally in animals[56]. Adverse energy is expected to affect brain function particularly: the brain is highly energy demanding, representing ~2–4% of body weight but consuming ~20% of the oxygen[57] and ~50% of the glucose[58]; and hippocampal vulnerability to energy depletion may make memory particularly vulnerable[34–36].

These findings expand adverse associations of dTFAs to health generally, and to central nervous system function in particular. Previously, dTFAs were adversely linked to behavior[28], and to mood[29]. The findings presented here add evidence for adverse associations to a third key prong of brain function—cognition. Findings cohere with previous findings linking food products that have *favorable* effects on oxidative stress, cell energy, and blood flow to *favorable* memory performance also confined to younger adult age[41].

This study has limitations. Data are cross-sectional, and unaccounted sources of bias and confounding cannot be excluded. However, documented adverse effects of dTFAs to mechanisms with documented relevance to brain function, including effects on essential fatty acids, oxidative stress, and cell energetics, offer material prospects for causality. This study used dietary report rather than objective markers of trans fatty acids such as red blood cell membrane trans fats[59], or plasma phospholipid trans fats[60]. This can be viewed as a strength or a limitation: while nutrient markers have the advantage of being objective, they have the considerable disadvantage that associations of nutrient *levels* to outcomes need not reflect any effect of either nutrient intake or nutrient levels *per se*, but may rather reflect effects of factors that modify the relation of nutrient intake to blood level[28]. For example, folate levels are influenced by genetic variants of methylenetetrahydrofolate reductase (**MTHFR**)—independent of intake[61]; and variants of MTHFR relate to numerous health conditions, in part through effects of MTHFR on DNA synthesis and DNA methylation[62]. Analogous processes could be in play for trans fat marker levels. Nonetheless, if further human studies of dTFAs were conducted, reassessment of the association using such measures would provide relevant triangulating information. dTFA was estimated from dietary recall. Not all foods that go by the same label have the same trans fat content. However, provided the misclassification is non-differential, this might be expected to produce bias toward the null (suggesting these findings may understate the true association). Although analyses that included the available women did not differ in findings, women were too few in number to draw separate conclusions. However, men represent half of the population, and a finding in this group is of independent importance. The study also did not include children, precluding conclusions about the dTFA-memory association in that group. Notably, though, the finding was stronger in younger individuals within the age group available for assessment.

This study also has material strengths. A randomized trial of dTFAs is unlikely to occur for ethical reasons (except perhaps of short duration), even if (or where) trans fats remain in the food chain. The window of opportunity for even observational assessment in humans may be closing, given the recent move of the USA Food and Drug Administration (**FDA**) to declare that trans fats are no longer “generally recognized as safe”[63]. This study occurred in a privileged window, after dTFA assessments had been added to the dietary assessment instrument, but before legislated trans fat labeling or restrictions were in place[64]—providing unusually favorable timing for examination of dTFA associations. Assessment of potentially important covariates, extending to exercise, mood and chocolate consumption, as well as evaluation of the full spectrum of metabolic markers, represents a key strength. The premise (and results) rest on a biological foundation. Factors including the relative strength of association, consistency of the main finding across a range of models bearing potential confounders, biological gradient (“dose response”), biological plausibility, and coherence with other literature were evident in our findings, and add weight to the possibility that the association we identify could have a causal basis.

### Implications

These findings, in which greater dTFA consumption is linked to worse word memory in adults during years of high productivity, adults age <45, add to evidence for unfavorable health correlates of trans fat consumption. They extend findings to a third pillar of central nervous system function, cognition—complementing evidence for adverse dTFA relations to behavior (aggression/irritability)[28] and mood[29]. Findings comport with recent FDA moves to rescind the designation as “generally recognized as safe” for dTFAs[65]; and add support to similar efforts in other nations.
